# Analysis of university students' participation in emergency education and its influencing factors in Shandong province

**DOI:** 10.3389/fpubh.2023.1097917

**Published:** 2023-03-09

**Authors:** Du Jie, Yang Mengzhe, Luo Huiwen, Lin Junchang, Zhang Yuhui

**Affiliations:** ^1^School of Public Health, Weifang Medical University, Weifang, China; ^2^School of Humanities and Social Sciences, Guangxi Medical University, Nanning, China; ^3^School of Management, Weifang Medical University, Weifang, China; ^4^China National Health Development Research Center, Beijing, China

**Keywords:** emergency education, training activities, to participate, Shandong province, university students

## Abstract

**Objective:**

The aim of this study is to understand the current situation of university students' participation in emergency education and its influencing factors in Shandong province, to improve the enthusiasm of university students' involvement in emergency training and exercise activities, and to provide a reference for universities to carry out education on public health emergencies.

**Methods:**

From April to May 2020, 6,630 university students were selected from six universities in Shandong province by stratified random sampling. Descriptive analysis, χ^2^ test, and logistic regression for statistical analysis were also used.

**Results:**

Overall, 35.5 and 55.8% of university students believed that it is necessary to participate in emergency education activities, and 65.8% of university students participated in emergency training and exercise activities. Through multivariate analysis, the results showed that university students who are men, sophomores, medical students, from within the province, the only child, have good health, take emergency education courses, think it is necessary to participate in emergency education, think the school attaches great importance to emergency education, believe that the professional teacher level to meet needs, know about public health emergencies, have received emergency education such as prevention and treatment of infectious diseases, and have a higher participation rate of emergency education and training activities.

**Conclusion:**

The willingness of university students to participate in emergency education in Shandong province is high, but the willingness in emergency training and exercise activities is low. Gender, grade, profession, and students' nationalities, whether it is only children, health, the school courses in emergency education situations, the value of emergency education, emergency education to participate, the degree of teachers' professional level to meet the requirements, a public health emergency condition as well as the prevention and treatment of infectious diseases such as emergency education are the main influencing factors for university students' participation in emergency training and exercise activities in Shandong province.

## Introduction

Major public crisis events caused by novel coronary pneumonia can seriously threaten the social order and harm human health (1, 2). As an essential part of the national emergency governance system, colleges and universities should be obliged to undertake the critical mission of safeguarding the lives and health of students and teachers and campus safety; however, with complex and mobile personnel on campus, since the emergence of the Omicron mutant strain, the risk of a cluster epidemic in schools has increased significantly. Emergency education refers to all emergency-related educational activities and behaviors in the field of emergency management. It is the educational activities and behaviors that teach all stakeholders emergency awareness, knowledge, skills, and values to prevent and respond to emergencies (3). In this study, emergency education for university students refers to the education to improve students' emergency literacy level to reduce the damage caused by emergencies *via* teaching them knowledge, skills training, and emergency drills. Colleges and universities as the main body to promote social development, college students as the main force to prevent and respond to emergencies, have an important influence on the overall prevention and control of emergencies and the effectiveness of their disposal as university students have a high demand for emergency education. The Emergency Response Law of the People's Republic of China stipulates that schools of all levels and types should incorporate emergency knowledge education into their teaching content. Schools should educate students on emergency knowledge and develop their safety awareness and ability to save themselves and each other (4, 5). Colleges and universities must assume the responsibility of emergency knowledge education for university students, build a better platform for their participation in emergency education, and effectively improve the emergency protection ability of university students in emergencies (6). The current research on emergency education mainly focuses on improving the emergency response system and emergency response capacity and studying countermeasures for emergency education in colleges and universities, which are macroscopic and lack targeted policy recommendations (7, 8). Therefore, this study mainly focuses on both macroscopic and microscopic aspects, and in the context of the new coronary pneumonia, it mainly investigates the participation of emergency education training and exercise activities of university students in Shandong province in public health emergencies and explores its influencing factors to put forward more targeted recommendations to improve the emergency literacy of university students and to better guide the emergency education work in colleges. This study conducted a questionnaire survey on public health emergency education in April–May 2020, using a stratified random sampling method to select 6,630 university students in six colleges and universities in Shandong province and reported the results as follows.

## Materials and methods

### Research subjects

The survey adopted a stratified random sampling method in April–May 2020 and divided Shandong province into three strata (comprehensive, science, and medicine), according to the nature of the disciplines. Each stratum randomly selected two colleges and universities, a total of six colleges and universities, based on the selected colleges and universities, and then according to the freshman, sophomore, junior, senior, and above grades stratified randomly selected university students for questionnaire survey. We collected 8,000 questionnaires, with 7,719 valid and an effective rate of 96.5%. Since some university students answered that they were unsure whether they had participated in emergency training and exercise activities, we excluded this sample size for rigorous consideration. The final sample size for analysis was 6,630. The ethics committee approved the study and obtained informed consent from the respondents.

### Research methodology

After coordinating and communicating with relevant departments and faculties of universities, we conducted a questionnaire survey on university students in anonymous form through the Questionnaire Star platform. The research contents include the basic information of the survey respondents, such as gender, age, ethnicity, and grade level, etc., as well as the awareness of university students about health knowledge and public health emergencies, university students' ability to cope with public health emergencies, the implementation of emergency education in schools, and the emphasis on health education and emergency education in schools. Before starting the survey, we clarified the questionnaire filling requirements and matters needing attention from the respondents. Each person can only answer the questionnaire once. Later, we sorted the submitted submissions and eliminated invalid questionnaires with missing basic information of survey respondents, incomplete answers to questionnaire entries, completeness rates < 95%, or response times of < 120 s.

### Statistical methods

Data were double-entered using Epidata 2.0, and SPSS25.0 software was used for statistical processing. We described count data as percentages, and multiple covariance tests were performed on the independent variables. We used the χ^2^ test and multi-factor unconditional logistic regression to analyze the influencing factors, with statistically significant differences at *P* < 0.05.

## Results

### Essential characteristics of the interviewed university students

A total of 6,630 interviewed college students were included. Most participants were men (65.1%), 19–21 years old (81.6%), Han Chinese (96.7%), freshmen (30.9%), and sophomores (24.6%). Most participants majored in science and technology (37%), and most participants were from rural households (68.8%), Shandong nationality (77.3%), and non-only children (63.5%), as shown in Table 1.

**Table 1:** Basic information of survey respondents.

**Variable**		**Number**	**Participation rate (%)**
Gender	Male	2,209	34.9
	Female	4,121	65.1
Age (years)	≦18	450	7.1
	19~21	5,168	81.6
	22~24	691	10.9
	≧25	21	0.3
Ethnicity	Han Chinese	6,124	96.7
	Ethnic Minorities	206	3.3
Grade	First-year	1,957	30.9
	Sophomore	1,559	24.6
	Junior	1,548	24.5
	Senior and above	1,266	20.0
Specialties	Medicine	1,789	28.3
	Literature and History	1,477	23.3
	Science and Engineering	2,340	37.0
	Art and Sports	724	11.4
Domicile location	Rural	4,352	68.8
	City	1,978	31.2
Place of origin	Within Shandong Province	4,896	77.3
	Outside Shandong Province	1,434	22.7
Is the only child	Yes	2,313	36.5
	No	4,017	63.5

### Knowledge of public health emergencies among university students

Among the 6,630 university students included in the study, 93% learned about public health emergencies through the Internet, TV, and radio; 4,169 of them knew what public health emergencies were, with a knowledge rate of 59.8%; 5,675 students knew about the generation and transmission channels of the new pneumonia epidemic, with a knowledge rate of 73.5%; 6,052 students knew about the protective measures of the new pneumonia epidemic, with a knowledge rate of 78.4%; 3,836 students knew the harmful effects of hepatitis B and other infectious diseases, with a knowledge rate of 60.6%; 3,826 students knew about the prevention measures of hepatitis B and other infectious diseases, with a knowledge rate of 60.4%; 5,440 students knew the correct way to wash their hands (seven-step method), with a knowledge rate of 85.9%; and 5,764 students knew the right way to wear a mask, with the highest knowledge and awareness rates of 91.1%. The knowledge of general first aid methods and emergency measures for things poisoning was the least satisfactory, with 56.6 and 53.5%, respectively.

### Receptance of emergency education among university students(includes science promotion and knowledge teaching)

Among the 6,630 university students interviewed, 4,883 had received emergency education related to infectious disease prevention and treatment. The receptance of emergency education about contagious disease prevention and treatment was 77.1%. A total of 2,703 students received emergency education related to accidental injury avoidance, and the receptance of unintentional injury-related emergency education was 42.7%. A total of 3,785 students received emergency education about earthquakes, typhoons, and other natural disasters, and the receptance of emergency education related to natural disasters was 59.8%. A total of 2,944 students received emergency education about fire, laboratory chemical leakage, and other accidents, and the receptance of accident and disaster-related emergency education was 46.5%.

### University students' willingness to participate in emergency education activities and participation in emergency education training and drill emergency training and exercise activities

Of the 6,630 university students interviewed, 2,358 students (35.5%) thought it was essential for them to participate in emergency education activities. Three thousand seven hundred five students (55.8%) thought it was necessary for them to participate in emergency education activities. One hundred forty-sevenstudents (2.3%) thought it was generally required for them to participate in emergency education activities, and only 1.9% thought it was unnecessary to participate in emergency education activities. Of the 6,630 university students interviewed, only 4,146 students said they had participated in emergency training and exercise activities, with a participation rate of 65.8%. The participation rate in emergency activities was much lower than the percentage of those who felt it was necessary to participate in emergency activities.

### Single-factor analysis of university students' participation in emergency training and exercise activities

Based on the differences in the primary conditions of individuals, university students' participation rates in emergency training and exercise activities differed by gender, age, ethnicity, grade, major, place of birth, whether they were only children, and health status. The differences were statistically significant (all *P* < 0.05).

Based on the influence of school level, the participation rates in emergency training and exercise activities of university students differed according to the availability of emergency education courses in schools, the degree of importance attached by schools, and the degree of teachers' professionalism to meet the needs. The differences were statistically significant (all *P* < 0.05).

Based on individual cognitive differences, the participation rates of emergency training and exercise activities of university students with different willingness to participate in emergency education and evaluation of emergency activities are different, also among university students with additional knowledge of public health emergencies, epidemic transmission routes, prevention and control measures, and acceptance of emergency education. The differences were statistically significant (all *P* < 0.05), as shown in Table 2.

**Table 2:** Participation in emergency training and exercise activities of university students with different characteristics.

**Variable heading**	**Variable classification**	**Number of people surveyed**	**Number of participants**	**Participation rate (%)**	***χ2* value**	***P-*value**
Gender	Male	2,209	1,656	75.0	127.966	< 0.001
	Female	4,121	2,506	60.8		
Age (years)	≦18	450	307	68.2	11.544	0.009
	19~21	5,168	3,423	66.2		
	22~24	691	422	61.1		
	≧25	21	10	47.6		
Ethnicity	Han Chinese	6,124	4,052	66.2	14.427	< 0.001
	Ethnic minorities	206	110	53.4		
Grade	First-year	1,957	1,356	69.3	54.034	< 0.001
	Sophomore	1,559	1,093	70.1		
	Junior	1,548	949	61.3		
	Senior and above	1,266	764	60.3		
Specialties	Medicine	1,789	1,267	70.8	75.482	< 0.001
	Literature and history	1,477	879	59.5		
	Science and engineering	2,340	1,479	63.2		
	Art and sports	724	537	74.2		
Domicile location	Rural	4,352	2,851	65.5	0.357	0.285
	City	1,978	1,311	66.3		
Place of origin	Within Shandong province	4,896	3,337	68.2	55.616	< 0.001
	Outside Shandong province	1,434	825	57.5		
Is the only child	Yes	2,313	1,617	69.9	27.994	< 0.001
	No	4,017	2,545	63.4		
Health status	Good	4,595	3,184	69.3	94.414	< 0.001
	Difference	1,735	978	56.4		
Emergency education course	Open	5,842	3,942	67.5	100.305	< 0.001
	Not opened	488	220	45.1		
Willingness to participate in emergency education	It is necessary	6,063	4,025	66.4	25.810	< 0.001
	Not necessary	267	137	51.3		
School importance	Very important	3,286	2,597	79.0	541.980	< 0.001
	General Importance	2,936	1,522	51.8		
	No attention	108	43	39.8		
The extent to which the level of teachers meets demand	Yes	4,522	3,309	73.2	387.612	< 0.001
	No	1,808	853	47.2		
Evaluation of the school's emergency activities	Very good	4,942	3,609	73.0	531.517	< 0.001
	General	1,243	502	40.4		
	Not good	145	51	35.2		
Public health emergencies	Understanding	3,902	2,938	75.3	411.509	< 0.001
	Don't know	2,428	1,224	50.4		
	Don't know	1,526	837	54.8		
Protective measures	Understanding	5,093	3,474	68.2	70.086	< 0.001
	Don't know	1,237	688	55.6		
Emergency education related to infectious disease prevention and control	Received	4,883	3,493	71.5	317.285	< 0.001
	Not accepted	1,447	669	46.2		
Emergency education for accidental injury avoidance and survival in hazardous environments	Received	2,703	1,907	70.6	48.282	< 0.001
	Not accepted	3,627	2,255	62.2		
Emergency education related to natural disasters such as earthquakes and typhoons	Received	3,785	3,141	64.0	31.238	< 0.001
	Not accepted	2,545	1,021	72.0		
Emergency education for fires, laboratory chemical spills, etc.	Received	2,944	2,059	69.9	42.876	< 0.001
	Not accepted	3,386	2,103	62.1		

### Multi-factor unconditional logistic regression analysis of university students' participation in emergency training and exercise activities

A multivariate logistic regression analysis was performed using 22 univariate factors with statistical significance, including gender, age, ethnicity, grade, major, place of origin, whether the student is the only child, health status, whether the school offers emergency education courses, and willingness to participate in activities. The VIF of all independent variables is < 10, and there is no multicollinearity. The dependent variable was whether university students had participated in emergency training and exercise activities (no participation = 0, yes participation = 1). The results showed that men, sophomores, medical, provincial students, the only child, good health, perception of the need to participate in emergency education, perception of the importance of emergency education at the school, perception of the professionalism of instructors as meeting the requirements, knowledge of public health emergencies, emergency education on infectious disease prevention and control, accidental injury avoidance, and perception of the importance of emergency education at the school. The percentage of participation in emergency training and exercise activities for university students in Shandong province was high, as shown in Table 3.

**Table 3:** Multi-factor unconditional logistic regression analysis of university students' participation in emergency training and exercise activities.

**Factors**		**Reference group**	**β**	***S.E***	**Wald*χ^2^* value**	***P*-value**	**OR value**	**95% *CI***
Gender	Male	Female	0.408	0.077	28.058	0.000	1.504	1.293~1.749
Grade	First-year	Senior and above	0.306	0.092	11.053	0.001	1.358	1.134~1.627
	Sophomore		0.406	0.092	19.349	0.000	1.501	1.253~1.799
	Junior		0.006	0.088	0.005	0.942	1.006	0.847~1.195
Specialties	Medicine	Art and sports	0.169	0.105	2.593	0.007	1.184	0.964~1.455
	Literature and history		-0.427	0.106	16.355	0.000	0.652	0.530~0.802
	Science and engineering		-0.366	0.099	13.642	0.000	0.694	0.572~0.842
Place of origin	Provincial	Out of province	0.431	0.066	42.582	0.000	1.539	1.352~1.752
Only child	Yes	No	0.199	0.059	11.350	0.001	1.221	1.087~1.371
Health status	Good	Difference	0.476	0.060	62.884	0.000	1.609	1.431~1.810
Emergency education course	Open	Not opened	0.265	0.127	4.335	0.037	1.304	1.016~1.674
Willingness to participate in emergency education	It is necessary	Not necessary	0.126	0.165	0.577	0.447	1.134	0.820~1.568
School importance	Very important	No attention	0.517	0.256	4.088	0.043	1.677	1.016~2.768
	General importance		0.094	0.251	0.141	0.707	1.099	0.672~1.796
The extent to which the level of teachers meets demand	Satisfaction	Unsatisfied	0.171	0.078	4.823	0.028	1.186	1.019~1.381
Public health emergencies	Understanding	Don't know	0.517	0.081	41.208	0.000	1.678	1.433~1.965
Emergency education for infectious disease control	Received	Not accepted	0.990	0.066	227.238	0.000	2.691	2.366~3.060
Emergency education for accidental injury avoidance and survival in hazardous environments	Received	Not accepted	0.154	0.068	5.173	0.023	1.166	1.021~1.332
Emergency education for fire and laboratory chemical spills	Received	Not accepted	0.174	0.065	7.159	0 0.007	1.191	1.048~1.353

## Discussion

Public emergency literacy is essential to emergency management, which can help the public effectively avoid risks (9–11). Recently, several school-related cases of the new coronary pneumonia (NCP) epidemic occurred in China. Because of the confined space and frequent personnel contact in schools, which create favorable conditions for virus transmission, schools have become a crucial and challenging point for preventing and controlling the NCP epidemic. Moreover, popular emergency education can effectively improve the emergency literacy of university students and help them better cope with the risk of epidemic normalization (12, 13).

The findings of this study indicate that university students' knowledge of the causes, modes of transmission, and protective measures for this new coronary pneumonia epidemic reached over 70%, but their familiarity with general first-aid techniques and emergency measures for things poisoning was < 57%. From this, it can be seen that under the influence of this new crown epidemic, university students' knowledge of public health emergencies, such as the transmission channels of infectious diseases, hazards, and preventive measures, has improved significantly. Knowledge of escape methods, first-aid skills for accidents and disasters such as fire, and emergency measures for public emergencies such as food poisoning is still unsatisfactory.

Second, the willingness of university students to participate in emergency education is generally high. Higher than the demand of university students in Liaoning province, 91.3% of university students in Shandong province believe that taking part in emergency education activities is necessary (11). However, the participation rate of university students in emergency training and exercise activities is 65.8%, which is still a big gap with 91.3% willingness to participate in emergency education. As a result, those university students who think it is necessary to participate in emergency education activities but have not participated in them should become the focus of emergency education in schools at the next step, so as to better realize the “unity of knowledge and action” and improve the enthusiasm of university students to participate in emergency training and exercise activities.

The multi-factor results showed that gender, grade, major, place of origin, whether the student is the only child, health status, school's emergency education courses offerings, school's attention to emergency education, university students' willingness to participate in emergency education, teachers' professional level to meet the needs, university students' knowledge of public health emergencies and the acceptance of emergency education courses are the main influencing factors for the participation of university students in emergency training and exercise activities in Shandong province.

The participation rate of male students in emergency training and exercise activities is higher than that of female students, 1.504 times that of girls and which is consistent with the findings of the study of Wang et al. (14). This may be due to the gender differences between male and female students, who tend to be more interested in emotional and linguistic cognition and less interested in emergency training and exercise activities that favor rational logic (15). The participation of freshmen and sophomores in emergency training and exercise activities is significantly higher than that of other grades, 1.358 and 1.501 times higher than that of seniors and above, respectively, and the proportion of sophomores participating in emergency training and exercise activities is the highest, probably because freshmen and sophomores are mainly studying relevant basic courses, and their academic pressure is relatively light, so they have more time and vigor to participate in social practice activities. The difference in the participation of junior, senior, and above students in emergency training and drills is not obvious because junior, senior, and above students have finished their basic courses and are in the critical period of internship, graduate school, and employment, so their time and experience are relatively scattered, and they are less motivated to participate in emergency education and training activities (16).

Students majoring in medicine are more likely to participate in emergency training and exercise activities than other majors, consistent with previous studies (17), maybe because medical students, influenced by the characteristics of their profession, have a greater love and respect for life. In addition, some medical schools offer specialized preventive medicine courses that can help medical students learn to pay attention to and participate in emergency training and exercise activities. The participation in the acceptance rate of education on public health emergencies among university students from inside the region is higher than that of university students from outside the province, which is 1.539 times higher, possibly due to the transformation of their living environments and cultural differences. University students from outside the province cannot quickly and steadily integrate into campus life. Weak campus integration leads to their limited participation in emergency education activities. Only children in emergency training and exercise activities are more than non-only children, 1.221 times higher than that of non-only children because only children bear more family responsibilities, which motivates them to participate in emergency education activities to better protect themselves and their family members. Health status affects university students' enthusiasm to participate in emergency training and exercise activities. University students with poor health status have a lower participation rate in emergency training and exercise activities, and the percentage of college students with good health status was 1.609 times higher than that of college students with poor health status. Due to the poor physical quality of this group, their ability to participate in practical activities related to emergency education is limited.

In addition, the degree of importance that schools attach to emergency education, the availability of emergency education courses, and the professionalism of school teachers to meet educational needs also affect the enthusiasm of university students to participate in emergency training and exercise activities. The importance of emergency education in colleges is 1.677 times higher than that of emergency education in colleges because the importance of emergency education in colleges will, to a certain extent, cause teachers and students to pay attention to emergency education, thus prompting college students to transform their ideological importance into behavioral participation and actively participate in emergency education and training activities. The participation rate of students in emergency training and exercise activities in colleges and universities with emergency education courses is 1.134 times higher than without emergency education courses because curriculum teaching is the most direct and effective way to improve emergency literacy, and starting emergency education courses provides an effective way for university students to learn about public health emergencies and related knowledge (18). At the same time, the professional quality of teachers and the degree to which they meet students' needs for emergency knowledge also implicitly influence university students' cognition and behavior in participating in emergency education and training activities.

University students with a higher willingness to participate in emergency education, knowledge of public health emergencies, and acceptance of emergency education on infectious disease prevention and control, accidental injury avoidance, survival in hazardous environments, and fire and laboratory chemical spills tend to have higher health literacy and better appreciate the hazards caused by public emergencies and the importance of improving their coping abilities, and as a result, emergency education training and exercise activities are more motivated, they are 1.304, 2.691, 1.166, and 1.191 times more likely to believe that it is not necessary to participate in emergency education and have received no relevant emergency education, respectively. Because individual perception is an important factor in promoting behavioral change, a strong tendency for active learning behavior and a strong sense of risk contribute to participation in emergency education and training activities. University students have a strong sense of risk, are susceptible to emergencies, and at the same time, they will recognize that there will be valuable consequences after the behavioral change, so they will take measures to improve their coping abilities, thus increasing their participation in emergency education and training activities. This increases their motivation to participate in emergency education and training activities (19).

To sum up, the participation rate in emergency training and exercise activities for university students in Shandong province is low, and the current situation of emergency education in colleges and universities needs to be improved. Colleges and universities should pay more attention to emergency education for university students to “prepare for rainy days and prevent problems before they occur.” Universities need to establish a long-term mechanism for emergency education, analyze the critical problems in emergency education, solve the contradictions in emergency education, and integrate emergency education into all aspects of education. The leadership of higher education institutions should pay attention to both thoughts and actions so that emergency education is not a mere formality (20). At the same time, university students are guided to raise their awareness of public health emergencies and to adopt a positive attitude and response. Moreover, the classroom content of emergency education should be tailored to the needs of students and be effective. Schools should hire more qualified teachers for emergency education positions and enrich their teaching work. Emergency education curricula can be combined with compulsory and elective courses to establish a curriculum system based on mandatory courses and supplemented by elective courses. In this way, the knowledge of emergency education can be comprehensively disseminated and meet the unique needs of students (21, 22). On this basis, colleges and universities should establish a perfect emergency disposal system, increase special investment in emergency education, regularly carry out emergency education and training activities, strengthen the practical exercises of emergency education for university students, and schools should plan a special emergency drill base and regularly carry out large-scale training and simulation drills (23, 24). Finally, the campus network, WeChat, microblogs, and other emerging media should be used to strengthen the daily publicity of emergency awareness and values among university students (25, 26), to guide them more positively, to explore more efficient, popular, and up-to-date forms of publicity, to create a healthy school environment and an atmosphere for preventing public health emergencies, to improve the emergency awareness of university students and to enhance their ability to respond to public health emergencies (27, 28).

## Conclusion

University students in Shandong province had a high willingness to participate in emergency education but low participation in emergency training and exercise activities. Men, sophomores, medical students, provincial students, only children, good health, and emergency education courses considered it necessary to participate in emergency education. The school attached great importance to emergency education, assumed that the professional level of instructors met the needs, knew about public health emergencies, and had received emergency education such as infectious disease prevention and control. University students' participation rate in emergency training and exercise activities is higher. Therefore, universities should raise the importance of emergency education for university students and carry out emergency training and exercise activities regularly.

## Data availability statement

The original contributions presented in the study are included in the article/supplementary material, further inquiries can be directed to the corresponding authors.

## Author contributions

All authors listed have made a substantial, direct, and intellectual contribution to the work and approved it for publication.
